# The New York Institute of Stomatology

**Published:** 1899-02

**Authors:** 

**Affiliations:** The New York Institute of Stomatology


### Reports of Society Meetings.

### THE NEW YORK INSTITUTE OF STOMATOLOGY.

A REGULAR meeting of the Institute was held Tuesday evening, October 4, 1898, at the residence of Dr. Benjamin Lord, No. 34 West Twenty-eighth Street, New York City, the Vice-President, Dr. C. A. Woodward, in the chair.

*Dr. Woodward.*—It is with great pleasure that the chair greets you, one and all, at this our first meeting of the season. It goes without saying that you have had a pleasant vacation, and I hope you have all come back with enthusiasm and determination to make this year a great one for this society. Last year was a good one, but it is believed that we can do better, and if we have the enthusiasm we ought to have, we will be able to accomplish it.

The minutes of the previous meeting of the Institute were read and approved.

*Dr. F. Milton Smith.*—The case which I present to the meeting to-night, and which some of you have seen, is an interesting one to me. Through an abnormal occlusion of long standing the patient, a lady of probably twenty-five years, has developed a deformity which detracts materially from the appearance of an otherwise pleasing face. Those of you who have seen it have noted that when the face is at rest the centre of the chin is thrown very much off the median line. There is also quite a depression on the left side of the face at a point opposite the upper canine tooth. An ineffectual attempt was made some years since to regulate the teeth, the deformity of the face then being not so apparent. I would be glad to receive suggestions looking to a correction of the deformity.

*Dr. J. Adams Bishop.*—There is only one suggestion that occurs to me, and that is to move the upper cuspid of the left side outward. As the mouth appears to me, I think it could be brought over with little trouble and would better balance the angles of the mouth.

*Dr. J. Morgan Howe.*—The case of irregular occlusion presented by Dr. Smith is an interesting one, and I think it points especially to the value of early intervention in cases of this kind. I suppose that the normal occlusion of the jaw must have been interfered with from the time of the development of the permanent teeth; that they developed in such relation as to throw the jaw over to one side. I have seen two or three cases like this in that respect. If the teeth had been interfered with, and placed in their proper position as they were developing, there would probably have been no difficulty, but I should suspect that after the jaw has been held over to one side for a series of years the temporo-maxillary articulation would be changed on both sides; that more or less thickening or deposition would change the shape of the glenoid cavities, and I should doubt very much whether interference with the position of the teeth at a late period would effect the desired change.

*Dr. George S. Allan.*—It is a case of double malformation. 1. The lower jaw is thrown out of its axial line so much as not to be accounted for by any jumping of the bite. This condition is congenital and cannot be remedied. 2. The articulation, owing to the contracted arch of the upper jaw, is altogether one-sided. This condition can be changed and the appearance of the face much improved; the articulation of the teeth at the same time being corrected.

*Dr. C. S. Stockton.*—I have a case which is certainly new and interesting to me. The patient is about thirty-five years of age. There was considerable swelling between the two central upper incisors, about equally distant between the gum margin and the apex of the roots. It was hard and had been very painful. I applied a capsicum plaster and had the patient call the next morning, thinking it a very simple and easy case to cure. It did not yield, and in a few days, probably a week, I concluded that the tooth was dead, although it gave no evidence of it whatever, every appearance being that of a live tooth except a slight soreness. However, I entered the tooth, found the pulp alive, and removed it, and thought there would be no more trouble; but, strange to say, it did not get well, and after waiting a sufficient time I removed the tooth. The gum-tissue was intact, there was no evidence of any disturbance except this swelling; when the tooth was removed the disease entirely disappeared. I would like to know what caused that. The patient healthy, the tissues intact all around the neck of the tooth, what should cause the caries of the root of the tooth as you see it?

*Dr. C. D. Cook.*—Caries or necrosis.

*Dr. Stockton.*—Caries I should say; if it had been necrosis I think it would have attacked the jaw, therefore I call it caries.

*Dr. W. St. George Elliott.*—I would like to state a case that came into my hands a few days ago, that of a gentleman about fifty years of age, teeth in good condition with the exception of the right upper lateral, which was abnormally white. I made up my mind that the tooth was dead; there was no apparent cavity in it, but merely the difference in color. On opening it from the palatine surface I immediately struck what appeared to be a living pulp, but, on further investigation, I found it was gum-tissue. In going farther I found that the whole of the interior of the tooth was filled with gum-tissue, and finally found a large cavity nearly a quarter of an inch in diameter below the gum, giving no external evidence of its presence whatever.

Dr. George L. Parmele, of Hartford, Conn., being introduced by the president, read a paper on "Tothe-Lore."

(For Dr. Parmele's paper, see page 1.)

*Dr. Elliott.*—In addition to what Dr. Parmele has told us, there are some interesting features of folk-lore in the East Indies. It is remarkable that the impression exists that pain in the mouth comes from a worm, remarkable inasmuch as it is general all over China, the Malayan Archipelago, and Japan. All through the East the dentist generally has his establishment on the sidewalk. If you were to go to a Chinese dentist with a toothache, he would say at once, "You have a worm in the tooth;" he would then examine your mouth, and in a few minutes he would bring out with his two chop-sticks a little worm; if the toothache was very bad he would introduce the chop-sticks again and take out another worm. The explanation is not difficult. On the side of the spatula there is a recess cut, a very shallow recess, and that is filled with artificial worms and covered with a thin piece of paper; when the spatula is put into the mouth the paper becomes wet and easily torn, and the worms escape into the mouth. It is an important fact that the Japanese are the only people in the world up to modern times who have done anything to speak of with artificial teeth. The Japanese, although they took their folk-lore and knowledge from the Chinese, have gone far beyond them. They can extract all the teeth and make artificial substitutes for both upper and lower jaws. They are of wood and remarkably serviceable.

*Dr. Robert H. M. Dawbarn.*—It has occurred to me that possibly Dr. Parmele might like to add to his list of Shakespearian folk-lore two more quotations of a very similar nature. In King Henry VI., third part, there are several such allusions. Dr. Parmele has mentioned one in Richard III. Two others are as follows in Henry VI. You remember the scene in which the humpbacked Duke of Gloster visits King Henry VII. in the tower and gloats over him, and the king, tormented beyond endurance, hits back by saying to Richard:

> "Teeth hadst thou in thy head when thou wast born,
> To signify, thou cam'st to bite the world."

Whereupon Richard, of course, up and kills him,—which was considered as being both an effective and a witty repartee in those days. But subsequently, in soliloquizing, he acknowledges the truth of the indictment as follows:

> "The midwife wonder'd; and the women cried,
> 'O, Jesus bless us, he is born with teeth!'
> And so I was; which plainly signified
> That I should snarl, and bite, and play the dog."

It is a fact that that same curious belief, that it is unlucky to be born with teeth, prevails in England to this day, and also in certain parts of this country, particularly where the descendants of English settlers are found.

Some years ago, in making some researches, I managed to collect as many as twenty-five indications of inherited syphilis, and the eminent surgeon Erichsen makes the statement that of such signs one is extremely early dentition; if that is the fact, then certainly early dentition would indicate bad luck.

I received a note from Dr. Howe a few days ago, asking me to discuss before you the subject of inflammation and suppuration, and saying that thirty to forty minutes were to be set aside for the purpose. I do not intend to talk to that extent, and the first member who wishes to intimate that he has heard enough will please yawn, and I will take the hint promptly.

The subject of inflammation and suppuration is as wide as pathology. One-half of all our general surgery is the surgery of pus, and the subject is practically limitless; any surgeon, I suppose, could talk all night upon it. It is merely a question of what you want to hear. Dr. Howe made a short list of subjects that he wanted touched upon; if there are in addition any others, and you will let me know, I will do what I can.

It appears to me that the points in relation to the subject that it would be most desirable for our members to listen to are the reasons for the differences often observed in severity and duration of the inflammatory process, the speedy termination, or, on the other hand, the long, protracted inflammatory condition. These are the points, I think, upon which some persons will wish to ask questions.

Now, suppose I take up these things in the order in which Dr. Howe mentioned them. First, the reasons for the difference in the severity and duration of inflammatory processes. I assume that by "inflammatory" the word "microbic" might be taken as a synonyme. "Inflammation," as a definition, as you know, is a word over which there has been any amount of discussion. "Inflammation" is now rather generally accepted as meaning a pathological process of microbic origin, and pathology may be defined as histology under difficulties. Now suppurative pathology is what you want discussed for the moment,—that is, the severity and duration of the inflammatory processes of a suppurative type.

There is hardly a microbe that is not capable of producing suppuration. Senn gives a list of ten mainly at fault, but any microbe is more or less of an irritant. The most common among these ten are, in severe suppuration, the streptococcus pyogenes, and in the milder, the staphylococcus pyogenes, of three kinds,—aureus, albus, and citreus. These four cover the great bulk of what we have to deal with. Now, you can tell before you see the patient, if you examine a drop of pus under the microscope, nine times out of ten, whether that patient is suffering misery or not. If I take a drop of the pus and stain it with methylene-blue, or other chemicals used for this purpose, and examine the little dotlets under the microscope, and find them clustered together in little masses or bunches, those are staphylococci pyogenes, and the patient is probably suffering very little; if, however, I find under the microscope apparently the same little dotlets, but arranged in strips, or bands, or chains, in a row, these are streptococci pyogenes, and I can be certain that the patient is having a rise of temperature, is unable to sleep at nights, and in considerable pain. The shorter the chains the worse the type of the microbe seems to be. A chain of three or four dotlets would thus indicate worse conditions than one of ten or twelve. As to the difference, then, in the severity of the inflammatory processes, they depend upon two factors: first, the kind of microbe causing it by the irritating presence of the chemicals called ptomaines and toxines, produced by its life and death processes; and, second, whether the inflammatory products are in unyielding tissues.

For example, of the five classes of "felons" (paronychia and conditions resembling it), the one that is the most agonizing is the one most deeply placed,—*i.e.*, under the periosteum. It is then the unyielding, inelastic character of the periosteum of the jaw that makes an immense difference in the amount of suffering, when pus forms beneath it.

As to the duration of the inflammatory suppurative processes, that depends upon quite a number of different factors. You all know that one of the modern names for the white blood-cells, which are little cannibals, guerilla warriors, is *phagocytes*, because they seek to devour the microbes. Wherever the microbes are found, there the white blood-cells accumulate in billions; and these immense cohorts of white cells attack the microbes wherever present and proceed to devour them. You can see under the microscope the microbes disappearing into the interior of these white cells until they cannot be followed farther, and presumably disintegrate. If the microbes get the upper hand, presently the white cells themselves die and disintegrate. Pus is a collection of such white blood-cells, plus the microbes, plus the "liquor puris," which is simply blood serum. In a case of suppuration, if you have only a few microbes present, it is quite conceivable that there may be a good deal of misery for a little while. If the microbes get the upper hand, and increase in numbers, then you begin to have a development of pus, and in ninety-nine times out of a hundred there is only one treatment for that pus, and that is to let it out; there is no medicine that will relieve it, and so long as it is left there, it is a steady trouble. If the microbes are present within bone in small numbers, and continue so, they are capable of raising a very slow and chronic form of inflammation, whereas, if they are present in large amounts in the bone, you have quite frequently *necrosis*,—that is, death of bone *en masse*, and promptly. Except in few instances, such as phosphorus necrosis, which is caused by a chemical irritant, in the great majority of cases, necrosis is caused by suppurative microbes, of any one of the numerous, different kinds. Now, if those microbes, instead of being in large amount, are only present in very small amount, and nevertheless the phagocytes cannot succeed in getting at them to destroy them, they may, by their irritating presence, induce a chronic osteitis. Their continued presence in the bone for months or longer is capable of exciting the growth of cells which are called "osteoclasts." These are a kind of "giant-cell," and wherever you have a chronic inflammatory process of absorption of bone going on they are found present, so that bone which was originally compact becomes more porous, and then that porous bone breaks down still further; and always in these chronic forms you find these osteoclasts present. After that porous bone becomes still more porous, comparatively large open spaces are formed in it, called the "caverns of Howship." Actual cavities in the bone of considerable size result from continued activity in this line. Often surrounding such examples of osteoporosis there will be found a zone of abnormally dense bone,—an osteosclerosis. The familiar case of Sir Benjamin Brodie, that famous English surgeon, is illustrative. He had a case once of a woman who came to him in great suffering and begged him to cut her leg off, saying she could no longer endure the pain. He finally amputated her leg at the knee, and subsequently, upon splitting open the tibia, found in it a chronic abscess,—a large, ragged cavity in the bone filled with pus. Of course, the correct treatment would have been to chisel into the cavity for drainage, and thus save the limb. Such an abscess is called to-day a "Brodie abscess,"—caused, as we have seen, by the presence of a few microbes, long continued, and exciting the activity of the osteoclasts.

As to the speedy termination of a long protracted suppurative inflammation, I think I have covered this.

Why does inflammation in the tissues contiguous to the bone sometimes terminate in necrosis, but more frequently does not?

I have also practically covered that point; it is a mere question of the number of microbes and their character, whether they are in unyielding tissue and whether they are so numerous that they can multiply readily. It is not the microbes themselves that kill the bone; it is the poisons they produce by their activities, and which also result from their death and decomposition.

I have been discussing necrosis. Caries is an absolutely distinct thing, and is almost invariably caused by the activity of the tubercle bacillus; so much so that caries of bone and tuberculosis of bone are now described as one and the same thing. In the matter of dental caries, that seems to be a disease by itself, and you, better than I, are able to determine what the cause of that is. With the exception of dental caries, caries differing from necrosis does not select dense bone; it will select the cancellous tissue of the tarsus and the bodies of the vertebræ.

Speaking of these suppurative microbes, there is one point that will interest you. There is a discussion that has never been settled as to whether the streptococcus of pus, this little microbe which is so extremely irritant, is or is not identical with the streptococcus of erysipelas. I have in my desk at home a letter from Professor Prudden, of Columbia University, taking the view that these microbes are identical, the clinical difference being probably that in the case of erysipelas the streptococci spread along the lymphatics, and in the case of suppuration, along the blood-vessels. Professor Welch, of Johns Hopkins University, takes the same view; but there are many pathologists who claim just the reverse,—for example Fehleisen, who thinks they are different microbes. It is one of the points in bacteriology to-day which ought to be cleared up, and in which there is much active work being done.

I need not say as to local indications, that nothing that you may apply to the outside, upon the skin or where it cannot reach the microbes directly, is going to scare those microbes. Still, in a certain way, but not by directly attacking those microbes, heat and cold have a therapeutic value. Every one knows that with a felon, for example, if treated locally (which it ought not to be except by the knife), the first thing usually is to apply cold, and then subsequently to apply heat as by a poultice. There is a reason for this, namely, that under cold the amœboid activity of the white blood-cells is very much retarded, and perhaps thus the formation of pus may be prevented. Now the amœba prima of ordinary ditch-water, if studied under the microscope, will move but sluggishly in cold water, but if the water is warm those primitive precursors of white blood-cells are very manifold more active. In other words, white blood-cells under heat can escape with great ease from the blood-vessels; but if you apply cold you check their activity. Therefore, when you have given up the hope of preventing pus and wish to bring a boil "to a head," you aid this by applying heat. But during the time that the medical man, as distinct from the surgeon, is applying his cold or heat the ptomaines and toxines from the microbes are poisoning the patient constitutionally, and threaten death locally. Therefore the knife, to give them free exit, is the logical remedy.

As to medicinal means, there is only one prominent medicine for checking amœboid activity, and that is quinine. All works upon therapeutics state that full doses of quinine will gradually diminish the tendency to suppuration. It does not, however, follow that you have helped your patient by preventing a tendency to suppuration, for, as stated heretofore, these accumulated leucocytes (white blood-cells, phagocytes) have power to attack and devour microbes.

Is there any other point now in connection with this subject? I think I have covered those that Dr. Howe asked me to speak on. Dr. Howe, in sending me this request, enclosed a brief history of a case in which there seemed to be a very unusually rapid suppuration. It is a case in which, if I understand it correctly, there was simply a discolored tooth containing a cavity; it had given no trouble whatever; he opened it to treat the discoloration, and simply found perfectly dry detritus. Having removed that, he treated the tooth with electrozone to bleach it and disinfect it, sealed it up, and for about twenty-four hours there was no discomfort; but pain appeared on the second day, and, the cotton being removed, a small flood of pus came out, showing that while there had been no discomfort whatever, the cleaning out of that detritus had, apparently, in some twelve to sixteen hours induced beginning suppuration, the microbes doubtlessly entering with the air. It seems to me likely that that suppurative activity had started pretty promptly after the operation. The electrozone had not been successful in disinfecting down to the bottom, and the suppurative activity had not reached the point at which it annoyed the patient until a number of hours had gone by. Now, this is a very interesting point, and I discussed it with various surgical friends of mine, none of whom could mention an authentic instance of pus-formation in less than twenty-four hours time after infection. I, myself, have never seen a case in which, although the wound might show every evidence of infection, actual pus appeared within twenty-four hours from the time of inception. I would simply suggest, not as a bleaching agent at all, but as a further antiseptic, the use of the formalin solution, or else, what I like very much in suppurating wounds, campho-phénique, which is nearly equal parts of camphor and carbolic acid (49 camphor, 51 phenol). It is unirritating; even if put on the skin it will not blister; and I think it might be a desirable thing to use in roots of teeth, much more so than electrozone would be.

*Dr. Howe.*—I am very much obliged to Dr. Dawbarn, and I am sure we all are. I am especially obliged to him for the care and attention that he has given to the points that I suggested to him, and he has remembered quite well the points of the case as I related them. I used electrozone because I wanted to bleach the tooth as well as disinfect it, it has effected both for me many times, but it did not answer in this case. I suspected, as soon as the patient reported the painful condition, that my electrozone had been kept a little too long. The point I wish to note especially was exactly what Dr. Dawbarn has stated,—that suppuration took place in a shorter time than I had ever known it to occur after the first indications of the disturbance, and I wanted him to tell in how short a time suppuration can result after inflammation begins. The first indications of the slightest discomfort were only sixteen hours from the time that I opened the root-canal and got a discharge of pus. When I opened the tooth it was entirely free from inflammation, and it never had caused any disturbance; the root-canal was entirely dry, and I did not disturb it more than half-way up the canal. That being Monday morning, it was Tuesday afternoon before the slightest sign of disturbance was recognized by the patient. I had cautioned him to notice carefully, and if there was the slightest discomfort to report at once. He did not come back, because it was so very slight on Tuesday afternoon, but Tuesday night it kept him awake, and on Wednesday morning, on removing all obstructions from the root-canal, I got a discharge through it of thick creamy pus. I would like to ask Dr. Dawbarn an additional question,—that is, to kindly give us more particularly than he did the clinical conditions as between necrosis and caries. That suggestion comes in connection with remarks that were made early in the evening.

*Dr. Dawbarn.*—I hardly know how to answer that, except, perhaps, by giving a typical clinical picture, and assume that you are discussing bones other than those of the teeth. I will give you one of the most common typical pictures of necrosis, then of caries. In the former we will say a child has been playing in the snow for a number of hours; he returns and goes to bed at night in the usual condition, but wakes up in the night in great agony. The pain is in a majority of cases in the shin; that is the most frequent seat of acute necrosis, somewhere near the knee. The agony may be so severe that the child is delirious, and he may hence even be unable to point out where the pain is. There have been all sorts of absurd diagnoses made in such cases. If nothing is done in the way of relief, the patient dies, or else in a few days pus appears at the surface, and one or a number of fistulæ form, which are technically called *cloacæ*, and which continue to discharge pus indefinitely. They lead down to dead bone, which is called a *sequestrum*. In about three months the sequestrum becomes loose, so that it can be detached from the living bone. This loosening is probably accomplished by the carbonic acid of the blood in its nascent form,—*i.e.*, just at the moment it is created,—which has a certain degree of solvent power upon dead bone.

As to the treatment of the kind of acute osteitis I have been discussing,—a kind which causes necrosis,—if seen early the knife and chisel will do wonders. No other treatment is worth discussing.

If you have made a mistake in diagnosis, your patient will not die from your chiselling into the bone; and if you have not made a mistake, you will have saved the patient either months of suffering or even his life.

If, however, the case is seen late and cloacæ have formed, the surgeon waits until the sequestrum separates, when he removes it by operation, and the remaining bone cavity may be treated in any of the modern ways. (Perhaps some of you remember my discussion of those a year or more ago before this Institute.)

Now a typical picture of caries. This is molecular death of bone, *not en masse.* In contrast with necrosis, a typical case of caries is extremely chronic. Assuming it to be in the tarsus, the child will limp a little; the foot will be somewhat swollen, as a rule, not red, but white. A common name is "white swelling." It will be worse when the patient's stomach is upset; it will be "barometric,"—*i.e.*, worse in bad weather. It will be at first more uncomfortable after rest; later, after exercise. Presently a point of redness will appear, and there will be a breaking down of the skin and exit of pus. If you were to run a probe into the fistula thus formed, you would probably be able to stick the end of it into soft bone; and the pus is often cheesy, occasionally gritty from bone-particles; and sometimes the probe, if left in for a while, will actually be discolored by sulphuretted hydrogen. The late Dr. Detmold used to say, *apropos* of these fistulæ, that anybody ought to be able to make a proper diagnosis in such a case without running in a probe or otherwise irritating it. The flabby, large granulations at its margin, the fact that a cold abscess is almost invariably tubercular (carious), and communicates with either a neighboring diseased bone or joint, suffices for an accurate diagnosis. The proper treatment is constitutional and local. The former includes cod-liver oil, milk, fresh air, and sunshine mainly. The latter either ignores the local trouble,—simply protecting the sore from trauma,—or else is as radical as possible, and removes every bit of the disease, scraping and gouging away the softened bone, and even cauterizing in addition. Iodoform is of great value in treating tubercular bone-troubles. Sterilized iodoform suspended in sterilized glycerin will often aid in curing them. Clinically there are two distinct types of caries at the two extremes of age,—viz., caries *suppurativa* and caries *sicca.* I have repeatedly seen on the soles of the feet of old people, or on the hands, chronic ulcers producing hardly any pus, but instead a bed of chronic granulation-tissue, which when scraped away exposes the carious bone from which it springs.

This is the so-called dry caries (*sicca*). The freely suppurating type—the kind causing much pus (suppuration)—we find mainly in the young. Psoas abscess is an instance of this,—an accumulation of pus and granular detritus caused by carious activity in the bodies of the vertebræ.

*Dr. Elliott.*—What do you think of the coal-tar derivatives as antiseptics?

*Dr. Dawbarn.*—Which of the coal-tar derivatives?

*Dr. Elliott.*—Antifibrin, for example.

*Dr. Dawbarn*—I presume we all know that this is identical with acetanilide. A higher price must be paid for the same drug if ordered under the trade name of *antifebrin.*

None of those coal-tar derivatives, while fairly good anti-rheumatics, antiseptics, and analgesics, are of particular value in tubercular troubles.

*Dr. S. E. Davenport.*—There is one point which I do not feel like starting a discussion upon to-night, but which seems to me to be a most important one. I remember having referred to it in a conversation with Dr. Howe, and, as he is chairman of the Executive Committee, I hope opportunity will be given at another meeting for an expression of opinion upon the subject. Is it advisable to remove from the canal of a tooth which is being opened for the first time, the pulp having died, the entire contents, or to remove a portion of the *débris* and then put in some germicide? Of course, it is a very old question, which has been many times considered; but we are all a little older now, and possibly some new thoughts may be brought forward on the subject. It has been the opinion of some, I know, that it is better to remove as much of the material as possible, for the sake of allowing the remedy to get to the point where it might be expected to do the most good.

Before sitting down I should like to propose a vote of thanks to our associate member, Dr. Parmele, who has come quite a distance, and who has entertained us most charmingly this evening.

The vote was unanimously carried.

*Dr. Smith.*—I was intensely interested in Dr. Dawbarn's talk. So many points were touched upon that I have nothing to say, other than to move a vote of thanks to Dr. Dawbarn for the very delightful talk that he has given us, and also to ask a single question of Dr. Howe. Dr. Dawbarn referred to formalin as being such an excellent remedy, as he thought, for cases of this kind. About eighteen months ago Dr. Howe read a paper before our society on this remedy, recommending it to us very highly, especially, as I remember it, in cases where the root had been newly opened. I should like to ask him what his success has been, and, if good, why he did not use it in this case?

*Dr. Howe.*—That is a very pertinent question. My success in the use of formalin has been very good, and I do not wish to retract anything I have said in its favor. I would, however, caution against using it in a strength more concentrated than five per cent., for I have found it a very great irritant, and capable of producing great pain. The only reason that I did not use it in the case under consideration this evening was that I wished to begin the bleaching of the tooth immediately, and I have had very good results from the use of electrozone in similar cases. I thought I would save time by combining the action of bleaching and disinfecting, which is practicable with this agent. I think my failure to disinfect the root contents in this case must have been due to using electrozone that had partially decomposed.

A vote of thanks was extended to Dr. Dawbarn, in accordance with Dr. Smith's motion.

Adjourned.

S. E. Davenport, D.D.S., M.D.S.,
*Editor The New York Institute of Stomatology.*

